# Shared Cultural History as a Predictor of Political and Economic Changes among Nation States

**DOI:** 10.1371/journal.pone.0152979

**Published:** 2016-04-25

**Authors:** Luke J. Matthews, Sam Passmore, Paul M. Richard, Russell D. Gray, Quentin D. Atkinson

**Affiliations:** 1 RAND Corporation, 20 Park Plaza, Suite 920, Boston, MA, 02116, United States of America; 2 School of Psychology, University of Auckland, Private Bag 92019, Auckland 1142, New Zealand; 3 Activate Networks, Inc., 1 Newton Executive Park, Suite 100, Newton, MA, 02462, United States of America; 4 Max Planck Institute for the Science of Human History, Kahlaische Strasse 10, D-07745 Jena, Germany; IFIMAR, UNMdP-CONICET, ARGENTINA

## Abstract

Political and economic risks arise from social phenomena that spread within and across countries. Regime changes, protest movements, and stock market and default shocks can have ramifications across the globe. Quantitative models have made great strides at predicting these events in recent decades but incorporate few explicitly measured cultural variables. However, in recent years cultural evolutionary theory has emerged as a major paradigm to understand the inheritance and diffusion of human cultural variation. Here, we combine these two strands of research by proposing that measures of socio-linguistic affiliation derived from language phylogenies track variation in cultural norms that influence how political and economic changes diffuse across the globe. First, we show that changes over time in a country’s democratic or autocratic character correlate with simultaneous changes among their socio-linguistic affiliations more than with changes of spatially proximate countries. Second, we find that models of changes in sovereign default status favor including socio-linguistic affiliations in addition to spatial data. These findings suggest that better measurement of cultural networks could be profoundly useful to policy makers who wish to diversify commercial, social, and other forms of investment across political and economic risks on an international scale.

## Introduction

Predicting how and why national outcomes vary is a major challenge for the social sciences. The political and economic behaviors of national states have profound consequences for the activities of businesses, universities, and nonprofits that operate within and increasingly across national boundaries [1]. However, while nation states are clearly a useful unit of analysis, they cannot be fully analyzed individually because they are involved in dynamic global relationships [2, 3]. Attempts to model national political and economic change have typically relied on various sets of predictor variables that are attributes of the countries sampled. Attributes used for prediction include GDP, temporal trend in GDP, infant mortality, outstanding debt, debt to GDP ratio, economic inequality, youthful age structure, and many other variables [4–13]. Other researchers have studied the potential effects of culture on national outcomes, but by operationalizing culture as a somewhat limited set of independent variables, rather than as a global network of historic and ongoing interconnections [14, 15]. Models built with these variables are used to predict political and economic outcomes such as the occurrence of protest movements, regime change, economic growth, and default on domestic or foreign debt.

The growth of online social networks, however, have rendered more visible the possibility that nation states are interconnected in essential ways [16]. The populations and leaders of nations influence each other through their actions, with the potential for countries to either adopt a behavior or decision based on other countries’ actions (cultural diffusion) or to react in an opposite manner (cultural differentiation). While the growth of online social networks has made national interconnectedness more apparent generally, researchers working with spatial models within both political science and economics have long recognized the role of such connections. Research in spatial econometrics has begun to address that spatial proximities between countries are not the most salient measure for the diffusion or differentiation dynamics [17–19]. For example, Asgharian et al. found that linkages of bilateral trade across countries better predicted co-movement in stock markets than did spatial proximity [20].

More recently, researchers have begun mapping global scale networks of how populations are interconnected socially. Much of this work has used email or social media data [16, 21]. Few, however, have examined how ancient patterns of cultural variation continue to structure social relationships at global scales or how they influence the transmission of contemporaneous cultural changes. For example, despite the potential for communication with anyone in the world, movements like the Arab Spring or #muslimrage spread within particular geographic regions where countries share close linguistic and religious ties.

Explicit measures of cultural similarity do not figure prominently in these models, probably because quantified cultural variables across many countries are difficult to find in the literature and it is often difficult to identify a principled set of cultural predictors *a priori*. Spolaore and Wacziarg dealt with the lack of good measures of cultural relatedness by examining the patterning of international differences in economic development on genetic divergence between countries at neutral genes. They found significant ‘genetic’ signal in cross-sectional tests of economic development, but interpreted these significant results arising because genetic divergence was a good proxy measure for inherited cultural beliefs and values [22, 23]. It seems similarly likely that observed spatial autocorrelations for political and economic characteristics are at least partly driven by spatial proximity being a proxy for cultural similarity. Thus, a more direct measure of cultural similarity may well improve quantitative models for important national phenomenon.

Indeed, a separate quantitative literature suggests that enduring cultural differences may play an important role in global geopolitics and economics. For example, broad civilizational affiliation of Yahoo email users predicts global emailing patterns in the present day [16], red and yellow carding of professional soccer players is substantially predicted by differences in their mother country’s cultural history of civil conflict [24], and cultural differences appear to drive variable behavior of study subjects during experimental economic games [25]. These findings on local scales or in specific contexts raise the question of whether macro-scale cultural variation predicts patterns of political and economic change in nation states around the globe.

### Language phylogenies as a proxy for cultural similarity

Cultural evolutionists seek to understand the fabric of cultural variation as the product of processes of cultural inheritance and diffusion. An increasingly important resource in this area are language family trees inferred through Bayesian phylogenetic analyses of cognate word lists [26–33]. Language family trees, or ‘phylogenies’ often trace the cultural ancestries of the groups who speak them and are sometimes presented as a paradigm example of species-like cultural evolution [32, 34]. Multiple lines of research indicate that language phylogenies are strong predictors of many cultural traits [28, 34, 35], suggesting that some elements of culture can be vertically co-inherited from ancestral populations along with language.

Much has been made of analogies (and disanalogies) between biological and linguistic or cultural evolution [32, 36–40], but languages need not be analogous to biological species for their phylogenies to exhibit coinheritance with cultural traits, and coinheritance is not the only socio-cultural process that would cause language phylogenies to predict cultural variations. Other socio-cultural processes may also create associations of various traits with language. For example, traits that diffuse by learning might spread more quickly across groups that have somewhat mutually intelligible languages (e.g. Spanish and Portuguese), than across groups whose languages are fully unintelligible to each other (e.g. Spanish and Finnish). Such a process could create a stepping-stone type diffusion that would result in an association of language phylogeny with certain traits even without classical co-inheritance.

A similar effect would occur if languages are themselves co-inherited along with core cultural beliefs and values that, in part, produce and maintain differences between cultures in how people interact, cooperate, perceive individual vs. collective rights, negotiate commerce and scarcity, etc. If core beliefs and values in turn influence cultural diffusion processes, then this could create a significant statistical association of the diffusing trait with language phylogeny. Such associations might be positive when core beliefs and values facilitate the diffusion process, but they might be negative if leaders or populations react against such diffusion. For example, an autocratic leader may crack down if protests spread among cultural relative nations, or leaders may act more fiscally disciplined if cultural relatives default on debt. In this manner, our hypothesized processes may influence many diffusing traits of contemporary practical importance.

Cultural core beliefs and values are often unspecified, difficult to measure operationally, and it can be difficult to determine what measurements of beliefs or values are relevant to predicting a particular outcome variable of interest.[41, 42] If language relatedness were a general proxy of these latent belief/value variables, then language phylogenies may have particular utility in applications to explain and predict the diffusion of behavior across countries.

## Methods

Here, we test this proposal using a cultural phylogeny of nations from the Indo-European language family and their associated colonies. As an outcome measure from political science, we used each nation’s “Polity score” in the Polity IV dataset, the most comprehensive and well-known scoring of the relative democratic or autocratic character of every nation for the past 200 years [43]. The Polity IV dataset was developed by the Political Instability Task Force for the purpose of finding exogenous variables that would predict complete collapse of state functions (‘state failure’). The dataset includes a combined Polity score that expresses on a 21 point scale how autocratic (-10) or democratic (10) a given nation state is.

As an outcome measure from economics, we considered national defaults (whether domestic or foreign) as reported in Reinhart and Rogoff 2011 [44]. Reinhart and Rogoff’s work is among the most comprehensive historical assemblages of default data at the national level. From their data, we constructed an outcome variable that classified for each year each country’s defaults to international or domestic creditors (S1 and S2 Tables). Because of the rarity of national default, we group years into 5-year bins starting from 1899. We then summed the defaults in each 5-year bin for each country.

In a similar way, from the Polity score we constructed a biennial dataset that included the Polity score for every other year starting with 1898 and going to 2012. We used two-year time bins because change in Polity score was more frequent than was domestic default.

### Autoregression as a method to investigate cultural transmission processes

We matched both sets of outcome data with location information and a matrix of socio-linguistic affiliations derived from the Indo-European language phylogeny [29] (see Construction of Socio-linguistic and Spatial Matrices) We included former colonies of Indo-European speaking countries when the colonial language was spoken by the majority of the population and no indigenous language was spoken by a majority. The distance to former colonies was set at the number of years since independence. This process results in a set of relationships that are not strictly bifurcating as in most phylogenies, and are not all directly tied to Bayesian phylogenetic estimates of divergence between distinct languages, since some distances come from colonial separations among populations. Therefore, throughout the paper we will refer to the relationships from this matrix as ‘socio-linguistic’. These distances are transformed (see Derivation of autocorrelation matrices from distance matrices below) into expected correlations among datapoints, which renders them valued (non-binary) ties in the terms of network science [45]. We call the resulting expected correlations based on language-colonial data ‘socio-linguistic affiliations.’ Similarly, we refer to expected correlations derived from the geospatial distances between countries as ‘spatial proximities.’

We sought to assess whether variation in Polity score or national default correlated across these socio-linguistic affiliations. To do this we used autoregression techniques implemented through the ‘lnam’ function of the R package ‘sna’ [46, 47]. Autorregression is a well-established technique from the spatial [48], social network [28], and phylogenetics [49] literatures used to assess whether a dependent attribute variable correlates across pairwise relationships among the data points in the model [50, 51]. We evaluated the performance of the socio-linguistic affiliations in concert with three other pairwise matrices, an ‘adjacency’ matrix of mutually intelligible languages, a spatial distance matrix, and a spatial ‘adjacency’ matrix of countries that share a border. Adjacency matrices are a standard representation from network science that encodes a 1 for all pairs that are connected together and a 0 for all pairs that are disconnected)[45]. Thus our language adjacency matrix encoded a 1 where languages are likely mutually intelligible, and our spatial adjacency matrix encoded a 1 where countries share a border.

We included the spatial data because prior autocorrelation modelling frequently has employed spatial data [48, 52, 53], most typically by using some form of spatial adjacency matrix [54]. Since spatial relationships are easily available, we wanted to assess whether the socio-linguistic affiliation matrix exhibited predictive utility beyond what was obtained through spatial analysis alone. Our rationale for including the adjacency matrix of mutually intelligible languages was similar. This matrix is easily available, so we wanted to asses if socio-linguistic affiliation showed statistical performance beyond what the language adjacency rendered. This also provides a strong test of our hypothesis that socio-linguistic affiliations reflect ongoing similarities in beliefs and norms that influence behavioral diffusion even across communities of distinctly different language speakers.

### Construction of the socio-linguistic and spatial matrices

We included in our analysis all nations from the POLITY IV dataset in which Indo-European languages were the majority language (e.g. English in England, French in France) or a recognized minority language with a history in the region (e.g. Kurdish in Iraq). Nations were matched with languages and the linguistic distance between the majority of languages was determined based on Bayesian phylogenetic inference from cognate data, following Gray and Atkinson [29]. Many of the languages in [29] were represented by more than one nation in the POLITY dataset. The lineages leading to these nations were treated as ‘dialects’ with divergence times based on historical evidence. For example, the diversification of European colonial dialects in the Americas was dated based on the founding of the colonies or the point at which they became independent. Likewise, the divergence (and occasional convergence) of the Italian and German states of the 20^th^ century was derived from historical records. Of course, it is not possible to precisely define a divergence point, but these dates provide approximate times that are adequate for the purposes of constructing the correlation matrices we use here. The socio-linguistic distance matrix and justifications for the timings used are provided in supporting information (S3, S9, S10 and S11 Tables, S1 File).

The spatial matrix comprised pairwise geographic distances between all nations in our sample, calculated using the Haversine formula. The location of each nation was recorded as its geographic centroid according to the CIA World Fact-Book [55]. These values were adjusted for Russia (and USSR) and Canada to represent more accurately the location of the bulk of the population and seat of power.

Spatial adjacency matrices were binary pairwise matrices with a 1 for countries that shared a land border and 0 for those that did not. Language adjacency matrices were set to 1 for countries whose languages were diverged by less than 1000 years, as this was a reasonable proxy for mutual intelligibility.

### Derivation of autocorrelation matrices from distance matrices

We converted the distances from the socio-linguistic and spatial matrices into an expected autocorrelation matrix with the following linear transform

$$\frac{\text{max}\left(D-D_{\text{ij}}\right)}{\text{max}\left(D\right)}$$

Where:

max(*D*) is the maximum distance value in the matrix,

*D*_ij_ is the distance observed between a pair *i* and *j*.

The rationale for this transform is it replicates the transform applied in a phylogenetic comparative approach that uses expected correlations derived from a bifurcating tree. Our set of socio-linguistic distances are mostly tree-like as well, and the cultural theory that underlies their use is closely related to this consistently validated approach of cultural phylogenetics [28, 32, 34–37, 56]. Additionally, a previous study of non-treelike genetic admixture in human populations empirically showed this transform performs reasonably well for autoregression models [57]. Spatial and language adjacency matrices were row-normalized as per the most common transform applied in network autoregression models [48].

### Statistical modelling

**Fig 1:**
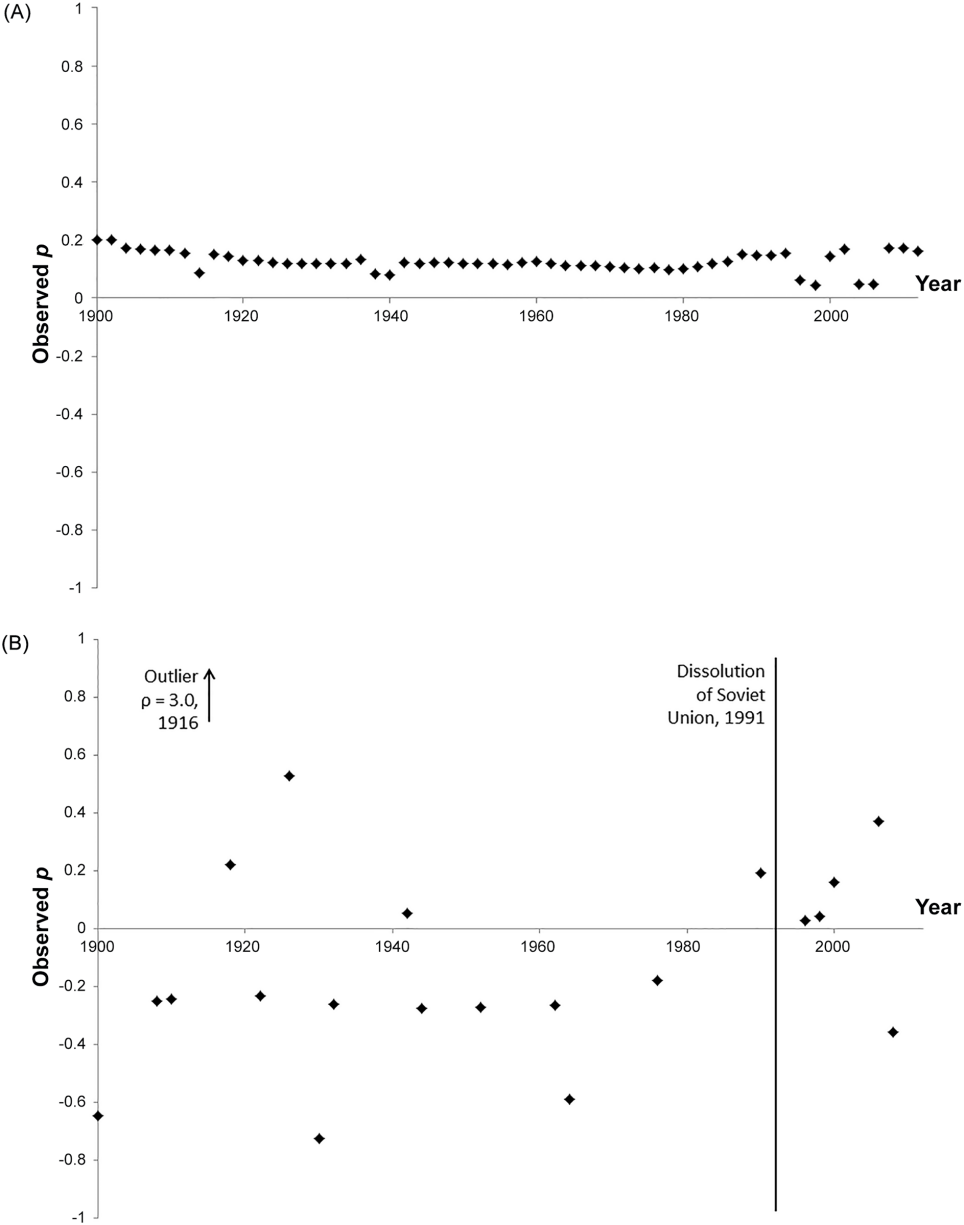
Autocorrelation of state (1a) and change (1b) in autocracy-democracy ‘Polity score’ on the socio-linguistic affiliation network at biennial intervals.

We conducted all statistical tests in the open source software R [47]. We used linear (Gaussian) autoregression models with the lnam function of the ‘sna’ package [46]. Our use of linear modelling for the dependent variables is obviously an approximation of the distribution of these outcomes, however, we think this approximation is preferable to other methods like splitting the outcomes into binary categories that would sacrifice information. While the outcomes are not distributed entirely normally, they do display a broad range of values over their distributions and over time (for polity score see figures from The Polity Project, http://www.systemicpeace.org/polityproject.html; for sovereign default see Reinhart and Rogoff Figure 1, available at http://www.nber.org/papers/w13882.pdf) [58].

The equation for the modelling of the state and change of Polity score can be written as:

Y=I+ε

ε=wε+v

Where *I* is the intercept, *Y* is the Polity score, and *ε* is the residual variation. The residual variation is itself split into two components, that which is correlated with the apriori autocorrelation weight matrices, and the independent and uncorrelated residual variation (*v*) [59]. An anonymous reviewer helpfully pointed out that the above equation for the residual term can be rewritten as:

$$\varepsilon =\frac{v}{1-w},\;\text{or as}\;\;\;\varepsilon \;\left(1-w\right)=\text{v}$$

which helps to make clearer the known property of autoregression that as w approaches zero the model becomes identical to ordinary linear regression with an uncorrelated residua l [48–51, 59]. Since *ε* is constrained by the empirical variation of the observed data compared to their predicted values from the independent variables (in our case the intercept, i.e. the mean) the quantity of uncorrelated residual (*v*) must decrease in proportion to the increase in autocorrelated residual (*w*).

The autocorrelation weight matrices are represented by *w*, which in the case of a model with all matrices is written:

$$w=\rho_{sp}W_{sp}+\;\rho_{sol}W_{sol}+\rho_{sa}W_{sa}+\;\rho_{la}W_{la}$$

Where *w*_sp_ is the matrix of spatial weights (proximities) derived from spatial distances, *w*_sol_ is the matrix of weights (affiliations) derived from socio-linguistic distances, *w*_sa_ is the matrix of spatial weights derived from spatial adjacencies, and *w*_la_ is the matrix of language weights derived from language adjacencies. The respective *ρ* values are the coefficients fit by maximum likelihood to these weight matrices [46].

The *ρ* coefficients are unbounded and estimated via maximum likelihood. There is no standardization of these coefficients as in regression on independent predictor variables because they represent the correlation of the outcome variable for each individual with the outcome variable for its network connections. A ρ = 1 can be interpreted that all the differences among the data points pattern as expected by the connections in the modelled network with more closely connected data points being more similar in outcome variable value, while a ρ = 0 indicates none of the observed differences pattern on the modelled network. Negative values indicate more closely connected values are more different in outcome variable value. Although ρ is commonly interpreted on a -1 to 1 scale [51], values less than -1 and greater than 1 are possible and reflect inflating the datapoint correlations predicted by the network.

### Simulation testing

Prior simulation research suggests that BIC modelling approaches demonstrate suitable statistical performance when used with the network autoregression model [60] however, other studies show that when autoregression is applied to some network topologies it may produce biased estimates for some parameters [61, 62].

Given this background, we conducted a set of simulations to assess potential biases present in the analyses on the networks particular to this study. To simulate diffusion on a network, we first generated a random normal variable for each node. We then added to this value 20% of the observed difference between this value and the average of their immediate neighbors in the network. We did this for 250 independently simulated variables on each of the four networks included in our study (spatial proximity, spatial adjacency, socio-linguistic affiliation, language adjacency) for a total of 1000 simulated variables. This simulation approach reflects a horizontal diffusion process in which all nodes simultaneously adopt a portion of the trait value of their network connections. We then processed these simulations with the same statistical approach as we used to analyze the empirical data.

## Results

### Socio-linguistic affiliation applied to the autocracy-democracy scale

Given the four matrices and possibilities for various combination models among them, we initially investigated preferred models through BIC model testing. All models regressed the Polity score on an intercept term and the matrices included in the given model. We did this for all possible model combinations across the Polity score for 58 biennial intervals (Table 1). The results indicate a median difference in BIC between the preferred and the 2^nd^ best model of 1.74, while a median difference of the 1^st^ and 3^rd^ most preferred of 3.53. We then considered how frequently each matrix was included in any of the top two preferred models, because the 3^rd^ model down is substantially worse than the 1^st^ preferred model (Bayes Factor of 7.06, strong support) [63]. The 1^st^ to 2^nd^ median Bayes Factor is 3.48 and is considered the lowest level of support, ‘positive’, in terms of Bayes Factors.

**Table 1:** Frequencies of models from BIC guided search of Polity Score.

	State of Polity Score		Change in Polity Score	
Model	Preferred	2nd best	Preferred	2nd best
L_adj_, SoL_aff_, S_adj_, S_prox_	1		1	7
L_adj_, SoL_aff_, S_adj_		6	1	2
L_adj_, SoL_aff_, S_prox_			4	3
L_adj_, S_adj_, S_prox_	7	6		
SoL_aff_, S_adj_, S_prox_	2	2		2
L_adj_, SoL_aff_	1	2	10	2
L_adj_, S_adj_		1	1	1
SoL_aff_, S_adj_	6	10	2	3
S_adj_, S_prox_			1	2
L_adj_, S_prox_	18	9		1
SoL_aff_, S_prox_	5	11	3	5
L_adj_		2	4	
SoL_aff_	18	9	7	8
S_adj_				5
S_prox_			7	11
null			16	5
frequency SoL_aff_ included	33	40	28	32
frequency L_adj_ included	27	26	21	16
frequency S_prox_ included	33	28	16	31
frequency S_adj_ included	16	25	6	22
	Overall		Overall	
frequency SoL_aff_ included	73		60	
frequency L_adj_ included	53		37	
frequency S_prox_ included	61		47	
frequency S_adj_ included	41		28	

L_adj_, = Language Adjacency (mutually intelligible), SoL_aff_ = Socio-linguistic affiliation, S_adj_ = Spatial Adjacency (shared border), S_prox_ = Spatial Proximity. For polity state 58 models were fitted while for polity change 57 models were fitted.

The top two most preferred models included the socio-linguistic affiliations 73 times, language adjacency 53 times, spatial proximity 61 times, and spatial adjacency 41 times. Interestingly, spatial adjacency alone was never one of the top two preferred models, nor was the combination of spatial adjacency with spatial proximity. Thus, spatial adjacency was only among the top two preferred models when in combination with some kind of language data. We think this indicates that at least some of the prior literature indicating spatial autocorrelation in Polity score occurred because spatial relationships in fact were a proxy for culture, which is better reflected by the socio-linguistic affiliation matrix.

As another test of the relative value of each matrix, we evaluated the number of significant autocorrelations when fitting each matrix individually. We found that polity score was significantly correlated (p < 0.05) across the socio-linguistic affiliation matrix in 58 of 58 intervals. Polity score correlated significantly across the spatial proximity matrix in 11 of the 58 intervals tested. Language adjacency alone correlated significantly in 51 of 58 intervals, while spatial adjacency correlated in 37 of 58 intervals.

Grouping together the statistically significant *ρ* values from the different time points, we found that the autocorrelation values for the spatial proximities were centered on zero (mean = -0.43, 95% conf. int. = -1.14 to 0.29, p = 0.21, one sample t-test, df = 10). In contrast, the parameter estimates for socio-linguistic affiliations were consistently greater than zero (mean = 0.13, 95% conf. int. = 0.12 to 0.14, p < 0.001, one sample t-test, df = 57, Fig 1a). Parameter estimates for language adjacency also were greater than zero (mean = 1.38, 95% conf. int. = 1.22 to 1.54, p < 0.001, one sample t-test, df = 50), as were those for spatial adjacency (mean = 0.43, 95% conf. int. = 0.41 to 0.46, p <0.001, one sample t-test, df = 36).

The above results indicate that countries with closely related languages or colonial histories tended to have similar Polity scores. This result does not appear to occur merely because socio-linguistic affiliation acts as a proxy index for spatial proximity or because some countries have mutually intelligible languages. While the language or spatial adjacency matrix considered independently show consistently positive autocorrelations, spatial adjacency is never among the top two models under BIC model selection, while language adjacency is only selected twice (Table 1). In contrast, socio-linguistic affiliation alone is the preferred model in 18 of 58 intervals tested, and it is the most frequently included matrix across the entire space of preferred models (Table 1). When modelled individually, only the socio-linguistic affiliation is significant in every interval. Considered overall, we think this indicates that socio-linguistic affiliation is the clear preferred matrix if a researcher were forced to choose one. The other matrices add explanatory power, but only when used in addition to the socio-linguistic affiliations.

### Socio-linguistic affiliation applied to change in polity score

**Fig 2:**
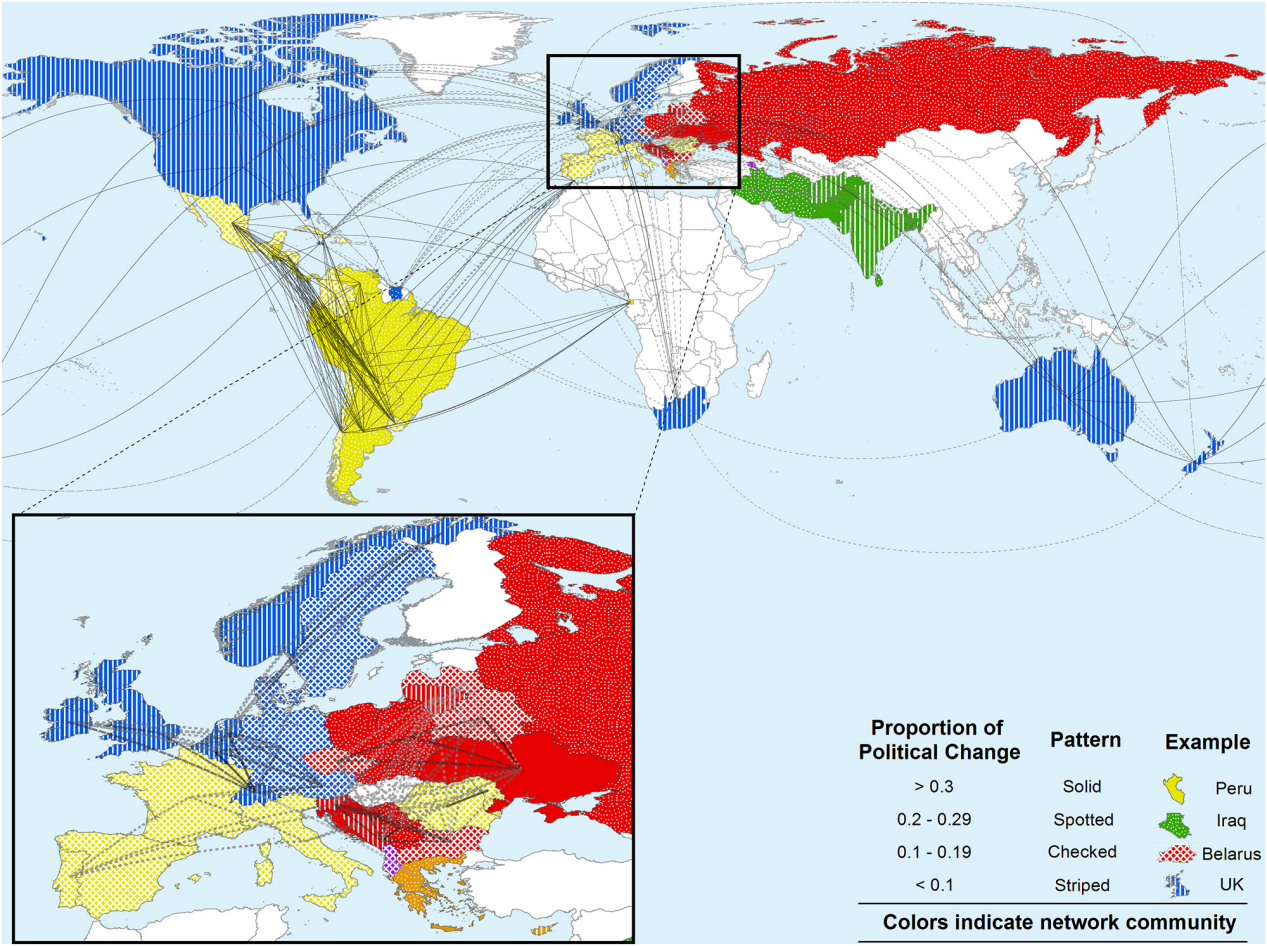
The density of political changes (proportion of intervals with any change in polity score) layered across geography and the socio-linguistic affiliation network. Network connection thickness is scaled to recency of ancestry. Only the 10 most recent connections are visualized for each country. Depicted network communities were inferred on the socio-linguist affiliation network through the Louvain algorithm [49] as implemented in Gephi [50].

We next tested whether biennial *change* in the Polity score was similarly correlated on the socio-linguistic and spatial matrices. We focused on biennial (every other year) tests because change in the fundamental character of a country (what Polity score measures) is quite rare (Fig 2). The limited number of changes over many annual periods would prevent any model fitting for these intervals.

Once again, a comparison of median BIC differences among the top three models indicated consideration of the top two preferred models (top models to 2^nd^ preferred—BIC difference = 2.22, Bayes Factor = 4.44; top models to 3^rd^ preferred BIC difference = 3.56, Bayes Factor = 7.12). Among the top two models, the socio-linguistic affiliation matrix was the most frequently included, and was selected in a model over twice as often as was language adjacency (Table 1).

Biennial change in Polity score was significantly correlated (p<0.05) across the socio-linguistic affiliations in 21 of 57 tests. The autocorrelations were significant on the spatial proximities in 11 of 57 tests. The statistically significant *ρ* parameters for change in Polity score were centered on zero for the socio-linguistic affiliation matrix (mean = 0.12, 95% conf. int. = -0.33 to 0.35, p = 0.94, one sample t-test, df = 20), but were significantly negative in the case of the spatial matrix (mean = -0.28, 95% conf. int. = -0.38 to -0.17, p < 0.001, one sample t-test, df = 10).

Parameter estimates for language adjacency were significant in only 10 of 57 tests, and were not statistically different from zero (mean = 4.79, 95% conf. int. = -6.07 to 15.65, p = 0.34, one sample t-test, df = 9). Spatial adjacency was significant in a mere 5 of 57 tests, and appeared centered on zero (mean = 0.29, 95% conf. int. = -0.08 to 0.66, p = 0.09, one sample t-test, df = 4).

These results indicate socio-linguistic affiliations was the most important matrix for predicting change in the Polity score variable. It is the most frequently selected in combination models, and it the most consistently significant matrix when considered singly.

### Socio-linguistic affiliation applied to sovereign default

We modelled the number of credit defaults by nation states on the socio-linguistic and spatial matrices. For the default outcome we used the sum of defaults both domestic or foreign [44]. The median difference in BIC between the top preferred models and the second preferred models was 1.87, while the median difference from the top models to the third ranked models was 2.64, which is a Bayes Factor of 5.3. As a Bayes Factor of 5.3 constitutes ‘strong’ evidence in favor of the preferred model [63], we consider the selection of models only among the top two contenders for each time period tested.

The spatial proximity and language adjacency matrices were the most preferred among the top two models for the 5-year time periods (18 cases each). Socio-linguistic affiliations were among the top two models in 11 cases, and the least preferred matrix at 7 cases was spatial adjacency (Table 2).

**Table 2:** Frequencies of models from BIC guided search of Sovereign Default.

	State of Sovereign Default		Change in Sovereign Default	
Model	Preferred	2nd best	Preferred	2nd best
L_adj_, SoL_aff_, S_adj_, S_prox_	1			
L_adj_, SoL_aff_, S_adj_			2	1
L_adj_, SoL_aff_, S_prox_		1	3	2
L_adj_, S_adj_, S_prox_				1
S-L_aff_, S_adj_, S_prox_				1
L_adj_, SoL_aff_	4	1	1	3
L_adj_, S_adj_	1	1		
SoL_aff_, S_adj_				
S_adj_, S_prox_		2	2	2
L_adj_, S_prox_	2	3	2	1
SoL_aff_, S_prox_	1			4
L_adj_	1	3	1	
SoL_aff_	1	2	4	1
S_adj_		2		
S_prox_	4	4	3	5
null	6	2	4	1
frequency SoL_aff_ included	7	4	10	12
frequency L_adj_ included	9	9	9	8
frequency S_prox_ included	8	10	10	16
frequency S_adj_ included	2	5	4	5
	Overall		Overall	
frequency SoL_aff_ included	11		22	
frequency L_adj_ included	18		17	
frequency S_prox_ included	18		26	
frequency S_adj_ included	7		9	

L_adj_ = Language Adjacency (mutually intelligible), SoL_aff_ = Socio-linguistic affiliation, S_adj_ = Spatial Adjacency (shared border), S_prox_ = Spatial Proximity. For default state 21 models were fitted while for default change 22 models were fitted.

In single matrix tests starting from 1904, the spatial proximity was significant only 1 time out of the 21 5-year time periods. Spatial and language adjacency were significant in 7 of 21 tests, while socio-linguistic affiliations were significant in 6 of 21. The statistically significant estimates for *ρ* were larger than zero for both language adjacency (mean = 0.2, 95% conf. int. = 0.06 to 0.35, one-sample t-test, df = 6) and socio-linguistic affiliation (mean = 4.20, 95% conf. int. = 0.65 to 7.75, one-sample t-test, df = 5) but appeared centered on zero for the spatial adjacencies (mean = -0.01, 95% conf. int. = -0.23 to 0.22, one-sample t-test, df = 6).

### Socio-linguistic affiliation applied to changes in sovereign default

We ran comparable tests for *change* in sovereign default by subtracting the number of defaults in one five year time period from the number of defaults in the next consecutive time period. Evaluation of the BIC scores in this case provide stronger evidence in favor of examining the most preferred model over the second ranked (BIC difference = 2.13, Bayes Factor of 4.26) but clearly support the preferred model over the third ranked models (BIC difference = 3.48, Bayes Factor of 6.96).

Considering the top two models, both spatial proximities (selected in 26 cases) and socio-linguistic affiliations (selected in 22 cases) are clearly preferred to the adjacency matrices that were each selected in fewer than 20 cases (Table 2).

Results from the tests of each matrix individually suggest that the dominance of spatial proximities and socio-linguistic affiliations during BIC selection comes from models in which they are combined with other matrices. Spatial proximity was significant in only 4 of 22 tests, while socio-linguistic affiliations were significant in 2 of 22 tests. In contrast, spatial adjacency was significant in 9 of 22 tests and language adjacency significant in 8 of 22 tests. Only language adjacency coefficients showed a significant difference from zero, which was negative (mean = -0.18, conf. int. = -0.35 to -0.02, one sample t-test, df = 7).

### Temporal effects on results

Our results focus on overall patterns from across the sampled time period in the patterning of outcome variables. If we tested for associations with independent attribute predictors then one might be concerned that any observed correlation actually is a spurious finding of temporal autocorrelation in the dependent and independent variables that are themselves unrelated [64]. That said, the concern may still arise that our findings come from time intervals that are temporally clustered, such as adjacent time points. The full set of models selected by year are in the supporting information (S4, S5, S6 and S7 Tables). We examined these models to assess whether key findings were robust to removing adjacent time points and only analyzing every other time point. Thus, key findings should be split evenly between the two alternative sets of non-adjacent time intervals (even and odd).

The key finding from our analysis of Polity score was that socio-linguistic affiliations were frequently included in models selected by the BIC method. Socio-linguistic affiliations were included in the top two models 73 times for the state of Polity score and 60 times for change in Polity score. When we considered only every other time point, socio-linguistic affiliations were within the top two preferred models for state of polity score on 53% of even intervals while this number for the change in polity score was 57%.

Regarding sovereign default, our key findings were models for the state of default tended to include language adjacency (18 times) and spatial proximity (18 times), while models for change in default included socio-linguistic affiliations (22 times) and spatial (26 times) proximities. When we excluded adjacent intervals from the state of default models, 39% of the selections for language adjacency occurred on even intervals, while this number for spatial proximity was 33%. Excluding adjacent intervals for the change of sovereign default showed that 36% of selections for socio-linguistic affiliations occurred on even intervals and 61% of selections for spatial proximities were on even intervals.

These analyses of alternate intervals suggest that the findings for Polity score are more robust to temporal clustering than are the results for sovereign default.

### Simulation testing

Our simulation tests produced median BIC differences that were very much in line with the BIC differences we observed in our empirical findings (Table 3). Depending on the simulated condition and models being compared, median BIC differences in simulations ranged from 1.19 to 3.88, while for the empirical tests they ranged from 1.87 to 3.56.

**Table 3:** Median BIC Differences from Fitted Models on Simulated Data.

Simulation Network	Preferred Model to 2nd Best	Preferred Model to 3rd Best
SoL_aff_,	2.18	3.73
L_adj_	1.19	2.82
S_adj_	1.31	2.67
S_prox_	2.51	3.88

L_adj_ = Language Adjacency (mutually intelligible), SoL_aff_ = Socio-linguistic (Language & Colonial History) Affiliation, S_adj_ = Spatial Adjacency (shared border), S_prox_ = Spatial Proximity.

We then examined the models selected amongst the top two preferred in a manner that exactly paralleled our treatment of the empirical data. The results showed that simulations on the networks of socio-linguistic affiliations or spatial proximities produced a preponderance of BIC-selected models that included these networks (S8 Table). For simulations on the socio-linguistic affiliations, the socio-linguistic affiliation matrix was included in selected models 460 times, with the next runner up being spatial proximities that were included 231 times. Similarly, when simulated data came from spatial proximities, this matrix was included in selected models 447 times with the runner up being socio-linguistic affiliations that were included 107 times.

Simulations on our adjacency matrices, however, indicated a degree of error in the models included among the top two preferred. Simulations on language adjacency were biased toward including spatial proximities in selected models (220 times) and selected language adjacency somewhat less frequently (189 times). Simulations on the spatial adjacency network included this matrix 226 times in selected models, but included the matrix of spatial proximities just as frequently (225 times).

## Discussion

These results indicate that socio-linguistic affiliations as measured by language and colonial relationships could be useful in risk mitigation models to the extent that they predict which countries will change in concert (up or down) in the medium-term future. The results are clearer for the Polity score variable since both BIC model selection and individual tests of statistical significance produce a clear signal that socio-linguistic affiliations are an important factor that structures how political change moves across the sampled countries. The results for sovereign defaults are more complex and showed more variation in result when we sampled non-adjacent intervals, but the BIC scores still suggest socio-linguistic affiliations are an important factor for use in combination with other matrices, particularly regarding changes in sovereign defaults over time.

The simulation results indicate caution is needed when interpreting selected models if both spatial proximities and either language or spatial adjacency are among the most selected matrices. This is because our simulations on adjacency matrices usually produced high rates of selecting the appropriate adjacency matrix, but also incorrectly produced high rates of selecting spatial proximities. Note, however, that high rates of incorrectly selecting socio-linguistic affiliations did not occur in any of the simulated conditions. The condition of concern arises in the empirical results only for the models selected for the state of the sovereign default variable, for which language adjacency and spatial distances were equally selected.

### Cultural trends through time for polity score

Since the socio-linguistic affiliation matrix was most commonly associated with significant Polity score changes (21 of 57 tests), we plotted the significant *ρ* over time. Although the average *ρ* across the sampled time was not significantly different from zero, we hypothesized that temporal trends may be apparent given the many geopolitical changes of the 20^th^ century. It would seem unreasonable to presume that all relevant socio-cultural dynamics of the system producing these correlations would remain constant over the 100+ year period we included in the analysis. We note an apparent clustering of significant and positive language autocorrelations during and after the end of the Cold War, for which the dissolution of the Soviet Union in 1991 serves as a reference point (Fig 1b). This may be a sequela from a less rigid geopolitical balance of power during and after the fall of the Soviet Union. This observation is consistent with some of Huntington’s ideas [65] that the end of a geopolitical Cold War among superpowers would make ancient civilizational affiliations all the more relevant to global politics. Our results are similarly consistent with recent observations of State et al. [16] that global patterns of email behavior show that language and Huntington’s civilizational categories remain critical predictors of who communicates with whom, even though in principal the internet allows users to communicate across traditional boundaries. Ironically, modern electronic communication may reinforce traditional cultural divisions by removing the geographic and governmental impediments that prevented individuals from choosing to communicate with those most like themselves. A tendency of people to preferentially associate with culturally or in other ways similar individuals is well established by the social network literature and is known as homophily [66].

### Reactionary dynamics for sovereign default

It is notable that the strongest signal for changes in sovereign default are significantly negative estimates of *ρ* across countries that speak the same or mutually intelligible languages. This is suggestive that countries who are recognizably cultural relatives to the world generally react against one another’s decisions regarding default, such that the default of one country may cause their language congeners to react by clearing away defaults. This result may be unexpected from general economic theory, which would predict default shocks to percolate first to countries that are the most closely linked economically and culturally. However, such reactionary effects are well-known in other domains of culture, such as linguistics, where social factors can create a push to increase language differences, particularly between neighbors or close relatives [67]—a process known as ‘esoterogeny’ [68, 69].

## Conclusions

Our findings reveal that the cultural ancestry of Indo-European nations, as documented by their linguistic relationships and colonial histories, carry important signals regarding contemporaneous political and economic changes. Even without predicting the occurrence or direction of change *a priori*, our results indicate that socio-linguistic affiliations might be used to better diversify investments or resources across countries that are less likely to change in concert. This application is analogous to the well-known stock diversification strategy of buying stocks across diverse sectors of the economy. The results suggest that autoregression modelling approaches of international-scale phenomenon can be improved by using socio-linguistic data in addition to the traditionally used spatial adjacencies.

Our findings provide strong justification for investment in more work on this area. This work could expand both the range of traits being examined and the range of cultural diversity. Our study used Indo-European speaking populations, but as researchers begin to produce dated language phylogenies for other language families [27, 30, 31, 70–72] and to explore deeper language relationships [73], there is the potential to apply these methods on a global scale. The approach outlined here also could be extended from political and economic variables to social phenomena as diverse as the spread of new products and technologies to the diffusion of social norms.

## Supporting Information

S1 File SplitJustifications. (DOCX)

S2 File FunctionSimCharOnNetworkContinuousModified. (TXT)

S3 File CodeNetworkSims7dec15. (TXT)

S1 Table ForeignDefaultFromWebsite. (CSV)

S2 Table DomesticDefaultFromWebsite. (CSV)

S3 Table LanguageDistances. Language File with Header Description.
(CSV)

S4 Table PolityState.by2Model. (TXT)

S5 Table PolityChange.by2Models. (TXT)

S6 Table SovDefaultState.by5Models. (TXT)

S7 Table SovDefaultChange.by5Models. (TXT)

S8 Table SimulationResults. (XLSX)

S9 Table LanguageDistances. Language File for Simulation Code.
(CSV)

S10 Table geo_adjacency_matrix. (CSV)

S11 Table Geographic_Distance. (CSV)
