# Supplementary material for: Shared Cultural History as a Predictor of Political and Economic Changes among Nation States
Source: PLoS One. 2016 Apr 25;11(4):e0152979. doi: 10.1371/journal.pone.0152979 (PMC4844133; doi:10.1371/journal.pone.0152979)
Supplement: S1 File — (DOCX) [file pone.0152979.s001.docx]

## Albania – Kosovo

- **Albania and Kosovo** have only split recently - within the last 5 years. Kosovo is still not a recognised state by all countries around the world.
  - Kosovo declared their independence from Albania in February 2008.
    - CIA. (2014). *The* *World Factbook – Kosovo.* Retrieved from <https://www.cia.gov/library/publications/the-world-factbook/geos/kv.html>

## Netherland – Orange Free State (OFS)

- **Orange Free State** was formed in 1854, following a treaty signed by the British. It ceased to exist in 1902 as the result of a treaty which ended the second Boer war.
  - - US Department of State: Office of Historian. () *A Guide to the United States' History of Recognition, Diplomatic, and Consular Relations, by Country, since 1776: Orange Free State.* Retrieved from <http://history.state.gov/countries/orange-free-state>

## Czechoslovakia – Czech Rep. – Slovakia

- Czechoslovakia was dissolved 21 years ago (1992) into the states Czech Republic and Slovakia
  - CIA. (2014). *The World Factbook – Czech Republic.* Retrieved from <https://www.cia.gov/library/publications/the-world-factbook/geos/ez.html>

## English Colonies

- **USA:** Europeans had started colonizing Canada & America by the early 1600's. USA was under British rule from 1770. We inserted a binary split from English_ST 237 years ago (1776) when America declared independence from Britain.
  - Library of Congress (1776) *Declaration of Independence.* Retrieved from [*http://www.loc.gov/rr/program/bib/ourdocs/DeclarInd.html*](http://www.loc.gov/rr/program/bib/ourdocs/DeclarInd.html)
- **Canada:** Europeans had started colonizing Canada & America by the early 1600's. Canada was under British rule from 1763. Canada declared independence in 1867. We inserted a polytomy in 1863 (150 years ago) for all of Canada, New Zealand & Australia. The difference between each of these country’s divergence from English culture was deemed to be negligible, which allows for the polytomy.
  - CIA. (2014). *The World Factbook – Canada.* Retrieved from https://www.cia.gov/library/publications/the-world-factbook/geos/ca.html
- **Australia:** Australian colonization began around 1788, with a settlement in NSW. Limited self-governance laws were passed in 1855. However, Australia remains a colony to Britain. Australia was placed in a polytomy with Canada & New Zealand 150 years ago, for reasons stated above.
  - Australian Electoral Commission*.* (2011)*. Australia’s major electoral developments timeline: 1788 – 1899.* Retrieved from <http://aec.gov.au/Elections/Australian_Electoral_History/reform.htm>
- **New Zealand:** Colonization began in 1828, with settlements in Keri-keri & Bluff. Self-governance laws were passed in 1852. NZ remains a colony to Britain. New Zealand was placed in a polytomy with Australia & Canada 150 years ago, for reasons stated above.
  - Taiwhanga, S. D. (1888). *The New Zealand Constitution Act – 15 and 16 Victoria, Cap. 72 Sec. 71.* Retrieved from <http://nzetc.victoria.ac.nz/tm/scholarly/tei-TaiProp-t1-g1-t1-body1-d8.html>
- **Trinidad & Tobago:** 1797, after a tumultuous history, Britain gained sole colonization of T&T. In 1962, T&T declared its independence from Britain. Trinidad & Tobago was placed in a polytomy with Jamaica and English_ST starting 51 years ago (1962). The independence of Jamaica and T&T was used as a point of divergence from English culture.
  - CIA*.* (2014). *The World Factbook – Trinidad and Tobago.* Retrieved from <https://www.cia.gov/library/publications/the-world-factbook/geos/td.html>
- **Jamaica:** Was under Spanish rule from 1509 – 1655, then British rule from 1655 – 1962. Independence was declared in 1962. Jamaica was placed in a polytomy with T&T and English_ST 52 years ago for reasons stated above.
  - CIA*.* (2014). *The World Factbook – Jamaica.* Retrieved from <https://www.cia.gov/library/publications/the-world-factbook/geos/jm.html>
- **Ireland:** *the first Norman-Irish lordship in Ireland (1177).* Irish culture begins to diverge from English culture with the Tudor conquest of Ireland in 1530 (483 years ago). *Independence from the United Kingdom in 1922.*
  - Irish Statute Book. (1922). *Constitution of the Irish Free State (Saorstat Eireann) Act*. Retrieved from <http://www.irishstatutebook.ie/1922/en/act/pub/0001/print.html>

## German States

- For the German states we have created a polytomy at 1230CE, the same point at which German splits from Dutch and the other lower saxon varieties.
- **Austria** & **Switzerland** split to independent branches that run to the present.
- **Baden, Bavaria, Saxony, Prussia** & **Wurttemberg** begin to diverge at the same time as Austria and Switzerland, continuing for around 650 years until 1871, at which time they join as the kingdom of Germany.
  - Carr. W. (1991) *History of Germany: 1815 – 1990*. London: Routledge, Chapman and Hall distributor
- **Germany** exists for a period of 78 years after the kingdom of Germany is established until the split of the state into **East** & **West Germany.**
  - Carr. W. (1991) *History of Germany: 1815 – 1990*. London: Routledge, Chapman and Hall distributor
- **East** & **West Germany** last for 41 years before the re-unification of **Germany** in 1990.
  - Carr. W. (1991) *History of Germany: 1815 – 1990*. London: Routledge, Chapman and Hall distributor
- **Germany** then exists for 23 years, which brings us to the present.

## Cyprus – Greece

- Cypriot culture is thought to have begun diverging from Greek culture after the establishment of the Cypriot state in 1832CE, following Greek and Cypriot separation from the Ottoman empire. Although the country was annexed by Britain in 1878 until 1960, it was largely uninfluenced by British traditions. This lead to binary divergence 181 years ago in our distance matrix.
  - Mallinson, B. (2005). *Cyprus: A modern history.* London: I.B. Tauris

## Italian States

- For **Modena, Papal States, Parma, Tuscany** & **the Kingdom of Two Scillies** we have a polytomy at 960 AD (1053 years ago). We use the “Placiti Cassinesi”, a group of four official juridical documents which are the earliest examples of written Italian (960AD) as an approximate date for the emergence of Italian dialects.
  - Fisher, J. H. (1986). European chancelleries and the rise of standard written languages. *Essays in Medieval Studies. Proceedings of the Illinois Medieval Association*, *3*(1986), 1-25.
- All states are then joined into the kingdom of Italy in 1870.
  - Stiles, A. (1989). *The unification of Italy, 1815-1870*. Hodder & Stoughton.

## Portuguese – Cape Verde

- Cape Verde was a Portuguese colony. The Island off the coast of Africa was primarily used for the slave trade and exile. A vicious civil war resulted in the country’s independence in 1975. We have thus entered a binary split at 38 years ago.
  - CIA (2014). *The World Factbook – Cape Verde.* Retrieved from https://www.cia.gov/library/publications/the-world-factbook/geos/cv.html

## Romania – Moldova

- Divergence from the Romanian culture is taken to begin in approximately 1940 with the establishment of the Moldavian Socialist Republic. We have inserted a binary split into our distance matrix at 73 years ago (1940) to quantify this divergence.
  - Mitrasca, M. (2002) Moldova: A Romanian Province under Russian rule: Diplomatic history from the archives of the great powers. New York: Algora Publishing.

## Russia – USSR – Kazakhstan

- The **USSR** was dissolved in 1991. From this we see the state of **Kazakhstan** established as well as re-instatement of **Russia**
  - We should note that we have data from Russia that predates the establishment of the USSR (1800 – 1922)
    - CIA (2014). *The World Factbook – Russia.* Retrieved from <https://www.cia.gov/library/publications/the-world-factbook/geos/rs.html>
    - CIA. (2014) *The World Factbook – Kazakhstan.* Retrieved from <https://www.cia.gov/library/publications/the-world-factbook/geos/kz.html>

## Serbo-Croatia

- The Serbo-Croatian region is an area of repeated social and linguistic divergence and recombination, making it difficult to represent as a set of pairwise differences.
- The state of Yugoslavia is also a problem; therefore we refer you to the image below for a visual explanation.
- We inserted a split at the year 1400 for **Serbia, Montenegro, Bosnia & Croatia.** There is evidence that the respective languages of these countries began to diverge around this time from numerous written legal and religious texts.
  - Petrovic, S. (2008). Oral and Written Art Forms in Serbian Medieval Literature. *Oral Art Forms and Their Passage Into Writing*, 85.
- Around 1990 we see the development of **Montenegrin**, a language very similar to Serbian, but culturally distinct.
  - Morrison. K. (2009). *Montenegro: A Modern history.* London: I.B. Tauris
- In 2002 we see the dissolution of **Yugoslavia** into the states of Bosnia, Croatia, Macedonia, Slovenia and Serbia & Montenegro. Bulgaria was never apart of Yugoslavia and therefore the cultural distance between Bulgaria and the remaining Serbo-Croation cultures is larger than the distance between each of the remaining Serbo-Croation cultures.
  - CIA. (2003) *The World Factbook – Yuogslavia.* Retrieved from <http://www.umsl.edu/services/govdocs/wofact2001/geos/sr.html>
- **Serbia & Montenegro**  then split in 2006 to form the independent states **Serbia** and **Montenegro.**
  - CIA. (2003) *The World Factbook – Serbia and Montenegro.* Retrieved from <http://www.umsl.edu/services/govdocs/wofact2003/geos/yi.html>
- **Bulgarian / Bulgaria,** although linguistically close to Macedonian, never joined Yugoslavia. Thus we see the skip over the Yugoslav line.

## South America

- Between 1811 and 1825, Spanish dominance in South America began to rapidly decline, with the independence of the countires listed below With differences in the time of divergence from Spanish being negligible between these states, we absorbed them all into a single polytomy 195 years ago (1818)
  - **Argentina (1818)**
  - **Bolivia (1825)**
  - **Chile (1818)**
  - **Gran Colombia (1819)**
  - **Mexico (1821)**
  - **Paraguay (1811)**
  - **Peru (1821)**
  - **United Provinces of Central America (1821)**
  - **Uruguay (1811 – 1830)**
    - Scheina, R. L. (2003). *Latin America’s Wars: The Age of the Caudillo, 1791 - 1899 – Volume 1.*  Washington D.C.: Brassey’s Inc.
    - Timeline of the Spanish American Wars of independence. (2011). *In Wikipedia.* Retrieved from <http://en.wikipedia.org/wiki/Timeline_of_the_Spanish_American_wars_of_independence>
- We then see some of the united states begin to dissolve into independent countries.
  - Gran Colombia was first in 1831 dissolving into:
    - **Colombia**
    - **Ecuador**
    - **Panama**
    - **Venezuela**
  - Resulting in a polytomy 182 years ago (1831)
    - Scheina, R. L. (2003). *Latin America’s Wars: The Age of the Caudillo, 1791 - 1899 – Volume 1.*  Washington D.C.: Brassey’s Inc.
  - United Provinces of Central America was next dissolved in 1840 resulting in the states:
    - **Costa Rica**
    - **El Salvador**
    - **Guatemala**
    - **Honduras**
    - **Nicaragua**
  - Again, this gave a polytomy, this time 172 years ago in 1840
    - Scheina, R. L. (2003). *Latin America’s Wars: The Age of the Caudillo, 1791 - 1899 – Volume 1.*  Washington D.C.: Brassey’s Inc.
- Spain held some states under its jurisdiction longer than the previously mentioned states
  - **The Dominican Republic** gained independence from Spain in 1865 (148 years ago), which was used as a point of divergence from Spanish culture.
    - CIA. (2014). *The World Factbook – Dominican Republic*. Retrieved from <https://www.cia.gov/library/publications/the-world-factbook/geos/dr.html>
  - **Cuba** gained independence in 1895 (118 years ago), we again used independence as a point of divergence from Spanish culture
    - CIA*.* (2014). *The World Factbook – Cuba.* Retrieved from <https://www.cia.gov/library/publications/the-world-factbook/geos/cu.html>
  - Spain’s African territory **Equatorial Guinea** declared its independence only 45 years ago in 1968. The independence of E.G was used as a point of divergence from Spanish culture.
    - CIA. (2014). *The World Fact-book – Equatorial Guinea.* Retrieved from <https://www.cia.gov/library/publications/the-world-factbook/geos/ek.html>
